# Variations and Predictors of Post-COVID Syndrome Severity in Patients Attending a Post-COVID Outpatient Clinic

**DOI:** 10.3390/jcm12124013

**Published:** 2023-06-12

**Authors:** Christina Lemhöfer, Thomas Bahmer, Philipp Baumbach, Bianca Besteher, Andrea Boekel, Kathrin Finke, Katrin Katzer, Katja Lehmann-Pohl, Jan-Christoph Lewejohann, Dana Loudovici-Krug, Matthias Nowka, Christian Puta, Stefanie Quickert, Philipp Alexander Reuken, Martin Walter, Andreas Stallmach

**Affiliations:** 1 Institute of Physical and Rehabilitation Medicine, Jena University Hospital/Friedrich-Schiller-University Jena, 07747 Jena, Germany; dana.loudovici@med.uni-jena.de; 2 Internal Medicine Department I, University Hospital Schleswig-Holstein Campus Kiel, 24105 Kiel, Germany; thomas.bahmer@uksh.de; 3 Airway Research Center North (ARCN), German Center for Lung Research (DZL), 35392 Grosshansdorf, Germany; 4 Department of Anaesthesiology and Intensive Care Medicine, University Hospital Jena, 07747 Jena, Germany; philipp.baumbach@med.uni-jena.de; 5 Department of Psychiatry and Psychotherapy, Jena University Hospital/Friedrich-Schiller-University Jena, 07747 Jena, Germany; bianca.besteher@med.uni-jena.de (B.B.); martin.walter@med.uni-jena.de (M.W.); 6 Department of Rehabilitation and Sports Medicine, Hanover Medical School, 30625 Hanover, Germany; boekel.andrea@mh-hannover.de; 7 Department of Neurology, Jena University Hospital/Friedrich-Schiller-University Jena, 07747 Jena, Germany; kathrin.finke@med.uni-jena.de; 8 Department of Internal Medicine IV (Gastroenterology, Hepatology and Infectious Diseases), Jena University Hospital/Friedrich-Schiller-University Jena, 07747 Jena, Germany; katrin.katzer@med.uni-jena.de (K.K.); matthias.nowka@med.uni-jena.de (M.N.); stefanie.quickert@med.uni-jena.de (S.Q.); philipp.reuken@med.uni-jena.de (P.A.R.); andreas.stallmach@med.uni-jena.de (A.S.); 9 Center for Sepsis Control & Care (CSCC), Jena University Hospital/Friedrich-Schiller-University Jena, 07747 Jena, Germany; katja.lehmann-pohl@med.uni-jena.de; 10 Department of Emergency Medicine, Jena University Hospital/Friedrich-Schiller-University Jena, 07747 Jena, Germany; jan-christoph.lewejohann@med.uni-jena.de; 11 Department of Sports Medicine and Health Promotion, Friedrich-Schiller-University Jena, 07747 Jena, Germany; christian.puta@uni-jena.de

**Keywords:** post-COVID score, prevalence, polysymptomatic, functional impairment

## Abstract

A relevant proportion of patients suffer from long-lasting impairments following an acute SARS-CoV-2 infection. The proposed post-COVID syndrome (PCS) score may improve comparison in the course and classification of affected patients. A prospective cohort of 952 patients presenting to the post-COVID outpatient clinic at Jena University Hospital, Germany, was enrolled. Patients received a structured examination. PCS score was calculated per visit. A total of 378 (39.7%) and 129 (13.6%) patients of the entire population visited the outpatient clinic two or three times, respectively (female: 66.4%; age: 49.5 (SD = 13) years). The initial presentation took place, on average, 290 (SD = 138) days after acute infection. The most frequently reported symptoms were fatigue (80.4%) and neurological impairments (76.1%). The mean PCS scores of patients with three visits were 24.6 points (SD = 10.9), 23.0 points (SD = 10.9) and 23.5 points (SD = 11.5) (*p* = 0.407), indicating moderate PCS. Female sex (*p* < 0.001), preexisting coagulation disorder (*p* = 0.021) and coronary artery disease (*p* = 0.032) were associated with higher PCS scores. PCS is associated with a multitude of long-lasting problems. The PCS score has proven its capability to objectify and quantify PCS symptoms in an outpatient setting. The influence of therapeutic measures on various PCS aspects should be the subject of further analyses.

## 1. Introduction

Although an infection with SARS-CoV-2 was initially regarded as an acute respiratory infection [1], it became obvious shortly after the start of the pandemic that a relevant proportion of patients develop long-lasting symptoms [2]. The prevalence of post-COVID syndrome (PCS) varies between different studies and ranges from 6% to 80% [3,4,5,6]. According to the WHO, the post-COVID conditions, also known as PCS, are defined as persistent or new-onset symptoms that are still present three or more months after the acute illness and that cannot be explained otherwise [7]. The frequency of a PCS is lower in vaccinated individuals [5] and in patients infected with less pathogenic variants [8]. However, the burden on affected individuals and health care systems is still high because of the increasing number of acute cases.

Until today, up to 200 different PCS symptoms have been described [6], including respiratory, psychiatric, cognitive, cardiovascular, gastrointestinal and inflammatory symptoms of different intensity, frequency and duration. The most frequent problems in affected patients are [9,10] fatigue, sleep disturbance, pain, cognitive dysfunction and/or depressed mood [11], and polysymptomatic disease in the vast majority of patients [9].

Despite the individual differences in symptom expression, patients generally suffer from a significant reduction in quality of life [3] and a reduced ability to work [12,13], underlining the need for both novel and individual therapeutic concepts and scoring systems to quantify treatment success. One possible scoring system for diagnosing and grading PCS was recently suggested by Bahmer et al. [14]. It was calculated in a population-based sample with ≥90% ambulatory disease histories. Therefore, applicability of the score in different countries and health care settings seems reasonable, as the frequency of inpatient treatments for acute COVID-19 in this study is similar to general population data from most other regions in the world. This PCS score, however, still needs to be validated longitudinally and in real-world data. Therefore, the aim of our study was (i) to analyze the severity of PCS in an outpatient cohort of a specialized post-COVID clinic, and (ii) to investigate the use of the proposed PCS score as a follow-up parameter. In order to take preventive action in patients at high risk in the future, we (iii) aimed to identify possible predictors for severe disease.

## 2. Materials and Methods

Patients presenting to the post-COVID outpatient clinic at Jena University Hospital between July 2020 and August 2022, and at least three months (84 days) after their documented SARS-CoV-2 infection, were included in the prospective registry. Infections with SARS-CoV-2 had to be detected by a positive PCR test or, if not available, by an antigen test before the appointment. Apart from these two specifications, there were no other exclusion criteria. The patients presented themselves in the outpatient clinic on their own initiative. Re-appointments were not scheduled automatically, but patients were encouraged to schedule a follow-up appointment if medically necessary. The interval between appointments varied as a result.

All patients underwent a structured assessment consisting of standardized questionnaires (Patient Health Questionnaire-9 (PHQ9) [15], Fatigue Assessment Scale (FAS) [16]), a cognitive screening test (Montreal Cognitive Assessment (MOCA) [17]), and screening instruments (Rehabilitation Needs Questionnaire (RehabNeQ)) [18] on self-reported post-COVID symptoms. In addition, pre-existing conditions were recorded in a structured manner, such as coagulation disorders, chronic lung diseases, or mental disorders. A physical examination also took place, the results of which are not part of this study. Further details of the enrollment process have already been described in Stallmach et al. [9], and are the same for this cohort. The respective viral variant was either taken from the direct microbiological results, if reported, or classified by infection date based on the Robert Koch Institute reports [19], i.e., the dominant variant was extracted from the reports for each week and aligned with the patient’s reported infection date.

The post-COVID score was calculated based on self-reported symptoms and screening results as reported by Bahmer et al. [14] for each visit. For this purpose, the presence or absence of each of the symptom complexes were assessed by evaluating the different subsymptoms. If one of the symptoms of the respective symptom complexes was present, the symptom indicator was multiplied with the individual point value of this complex, ranging from 2 to 7. All point values of the single symptom complexes are summed up to the total score. The PCS score consists of 12 self-reported, non-overlapping symptom complexes in total, and was developed by a hypothesis-free clustering procedure employing k-means clustering, amongst others. Through this clustering procedure, each symptom complex is assigned an individual point value indicating its contribution to the total severity of PCS. In brief, a score of 0 points indicates absence of PCS, scores below 10.75 points correspond to mild PCS, scores between 10.75 and 26.25 points indicate moderate PCS, and scores above 26.25 indicate severe PCS [14].

### Statistical Analyses

In a descriptive manner, the mean and standard deviations for numerical variables are reported. For categorical variables, absolute and relative frequencies (%) are mentioned. In the case of exploratory comparisons, the chi-square test was used for categorical variables and, respectively, the *t*-test for numerical variables. For comparisons between three groups with different treatments in the acute phase, an ANOVA with post-hoc tests (Bonferroni-adjusted) was used. Pearson correlation coefficients were used to calculate the association between different variables. A two-sided significance level of *p* < 0.05 was applied. Statistical analyses were performed using SPSS (v29, BM Inc, Armonk, NY, USA).

## 3. Results

### 3.1. Patient Demographics

#### 3.1.1. First Presentation (FP)

**Figure 1:**
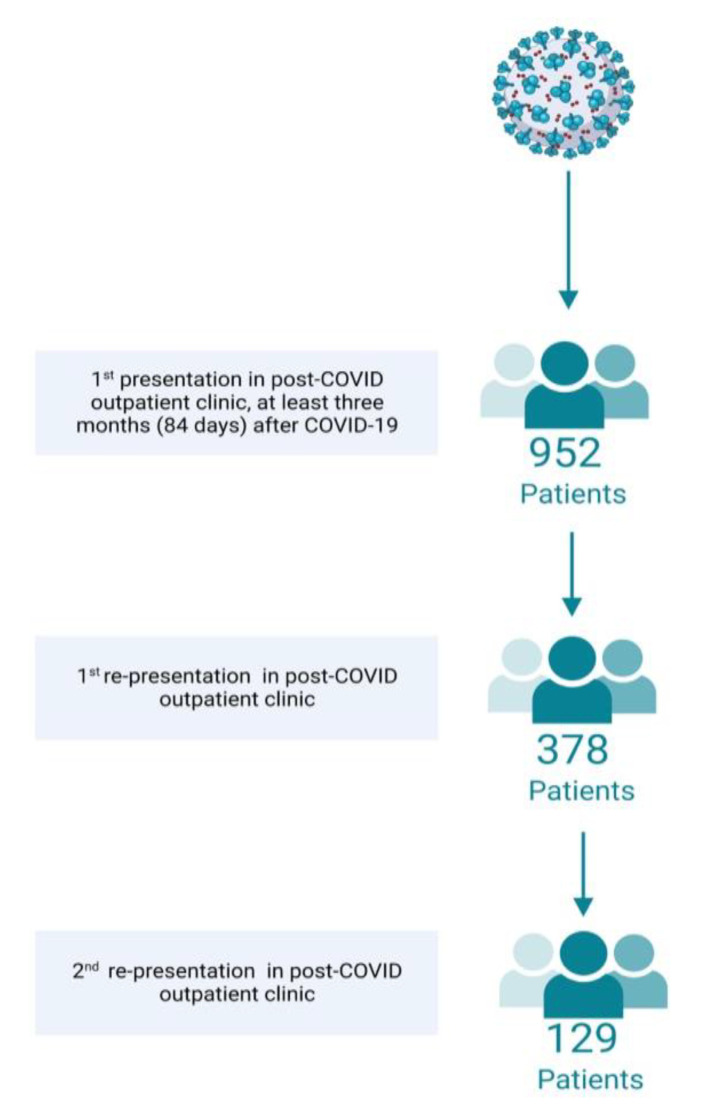
Flowchart of patients enrolled (Created with BioRender.com).

A total of 952 patients were included in the analysis (Figure 1). Of these, 632 (66.4%) were female. The mean age was 49.5 years (range = 19–91 years; SD = 13). Mean BMI was 27.9 kg/cm² (range = 16.1–56.0 kg/cm²; SD = 6.0 kg/cm²).

On average, FP took place 290 days (range = 84–832 days, SD = 138 days) after the SARS-CoV-2 infection. The exact interval between infection and FP are depicted in Table 1. According to the time of infection, it can be presumed that 89.0% (*n* = 847) were not infected with a virus variant of concern. 5.6% (*n* = 53) were infected with the alpha (B1.1.7), 3.5% (*n* = 43) with the delta, and 0.9% (*n* = 9) with the omicron variant (BA1). The results of the questionnaire-based assessments revealed that 13.9% (*n* = 127) of the subjects had no depression, 29.1% (*n* = 266) had mild depression, 31.9% (*n* = 292) had moderate, and 25.1% (*n* = 230) had severe depression, according to the PHQ9. Fatigue could be confirmed by means of the FAS in 83.8% (*n* = 763). Mild cognitive deficits could be objectified using the MOCA in 33.7% (*n* = 253). One person had severe cognitive deficits (0.1%) and in 496 (66.1%) no general cognitive deficit was detected by the broad cognitive screen.

Pre-existing conditions, as well as treatment during the acute phase, are shown in Table 1.

#### 3.1.2. First Re-Presentation (1st RP)

A total of 378 patients (39.7%) presented again to the post-COVID outpatient clinic. The mean duration since infection was 393 days (range = 133–882 days; SD = 123). The exact duration is depicted in Table 1. Of these patients 248 (65.6%) were female.

#### 3.1.3. Second Re-Presentation (2nd RP)

Within the 129 (13.6%) affected individuals who presented a third time, the acute infection had occurred on average 506 days (range = 296–860 days, SD = 114) previously. Table 1 depicts the differences in duration since infection. 85 of these patients (65.9%) were female.

### 3.2. Post-COVID Score

Considering all included patients, the mean post-COVID score was 20.9 points (SD = 10.4) at the time of FP, 22.3 points (SD = 11.5) at the 1st RP, and 23.5 points (SD = 11.5) at the 2nd RP. In the subcohort of patients with all 3 outpatient visits, the post-COVID score was 24.6 points (SD = 10.9), 23.0 points (SD = 10.9), and 23.5 points (SD = 11.5). We found no significant differences between the time points (repeated measures ANOVA; sphericity assumed, F(2, 256) = 0.903; *p* = 0.407; partial ɳ^2^ = 0.0007).

**Figure 2:**
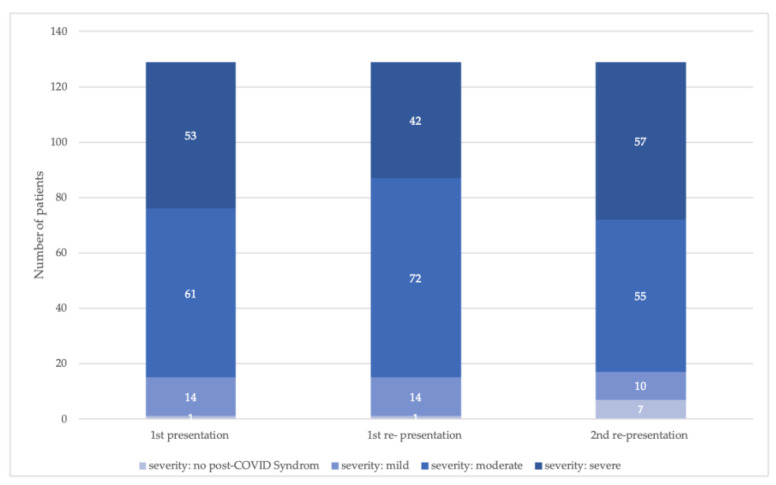
Frequency of post-COVID score-defined severity classes at the time points of presentation, focusing on patients who presented on each time point (number of patients = 129).

The corresponding severity of PCS for those who presented three times is shown in Figure 2 and the frequency of existing symptom complexes at the time points of consultation in Table 2.

At the time of FP, fatigue (80.5%), neurological ailments (76.1%), and exercise intolerance (53.8%) were the most common health conditions. At 1st RP, fatigue (80.2%) and neurological ailments (76.5%) were also frequently reported. The third-most frequently reported symptom complex was joint and muscle pain (54.4%). This remained unaltered at the 2nd RP, with only slight changes in relative frequency: fatigue (82.2%), neurological ailments (80.6%), and joint and muscle pain (55.5%).

### 3.3. Post-COVID Severity at the First Presentation: Associated Variables

Females (M = 22.2 points; SD = 10.5) showed a significantly higher post-COVID score compared with males (M = 18.4 points; SD = 10.0) (*t*(950) = 5.28; *p* < 0.001). When considering the pre-existing conditions, self-reported coagulation disorder (existing M = 25.6 points; SD = 8.5, non-existing M = 21.0 points; SD = 10.4; *t*(911) = −2.31; *p* = 0.021) and coronary artery disease (existing M = 18.2 points; SD = 10.5, non-existing M = 21.2 points; SD = 10.3; *t*(910) = 2.15; *p* = 0.032) showed significant differences PCS severity groups. No differences were found for chronic lung diseases (*t*(917) = −0.774; *p* = 0.439), chronic heart failure (*t*(911) = 1.824; *p* = 0.068), chronic pain (*t*(910) = −1.05; *p* = 0.295) or mental disorders (*t*(912) = −1.31; *p* = 0.191).

Results showed no correlation between post-COVID score and age (*r*(952) = −0.001; *p* = 0.985) or BMI (*r*(938) = 0.004, *p* = 0.914). Days since infection (*r*(952) = −0.083, *p* = 0.011) also did not correlate with post-COVID score.

There was a significant difference in the post-COVID score in patients treated as outpatients (M = 21.1 points, SD = 0.5), inpatients (M = 21.8 points SD = 10.1), or critically ill patients (M = 17.6 points, SD = 10.6; F(2) = 4.860; *p* = 0.008). In the post hoc comparisons, significant differences were revealed between outpatient versus intensive care treatment (M_Diff_ = 3.55; *p* = 0.011) and inpatient versus intensive care treatment (M_Diff_ = 4.22; *p* = 0.010).

Further analysis showed that there was a modest correlation between the level of depression (PHQ9) (*r*(907) = 0.316; *p* < 0.001) and fatigue (FAS) (*r*(900) = 0.303; *p* < 0.001) and the post-COVID score. However, there was no correlation between the cognitive screening score (MOCA) and the post-COVID score (*r*(744) = −0.004, *p* = 0.914).

#### Acute Treatment Setting, Post-COVID Symptoms and Time since Infection

Further analyses revealed significant differences comparing the treatment modality during the acute phase with respect to the presence of certain symptom complexes. This was the case for the symptom complexes fatigue (outpatient = 81.3%, inpatient = 83.8%, critically ill = 67.1%; *Χ*^2^(2, *n* = 952) = 10.71; *p* = 0.005), cough (outpatient = 12.0%, inpatient = 21.6%, critically ill = 9.8%; *Χ*^2^(2, *n* = 952) = 10.73; *p* = 0.005), neurological ailments (outpatient = 78.1%, inpatient = 75.0%, critically ill = 59.8%; *Χ*^2^(2, *n* = 952) = 13.74; *p* = 0.001), and chemosensory deficits (outpatient = 34.4%, inpatient = 24.3%, critically ill = 13.4%; *Χ*^2^(2, *n* = 952) = 15.04; *p* < 0.001).

A significant association was found between treatment modality and days between infection and FP at the post-COVID outpatient clinic. Critically ill patients presented rather shortly after the infection (M = 230 days, SD = 116), followed by inpatients (M = 275 days SD = 145) and outpatients (M = 301 days, SD = 137; F(2) = 11.179; *p* ≤ 0.001). The post hoc comparison demonstrated significant differences only for outpatients versus patients with intensive care treatment (M_Diff_ = 71; *p* ≤ 0.001), but not for inpatient versus outpatient treatment (MDiff = −26; *p* = 0.093) or inpatient versus intensive care treatment (MDiff = 45; *p* = 0.056).

### 3.4. Frequencies of Re-Presentation

Patients who presented only once (*n* = 574) to the post-COVID outpatient clinic were 46.5 (SD = 12.3) years old, and had a BMI of 26.9 kg/m² (SD = 5.5). Re-presenters (*n* = 378) were significantly older (M = 54.12, SD = 13.6; *p* > 0.001) and had a significantly higher BMI of 29.5 (SD = 6.3, *p* < 0.001). We found significant sex differences between patients presenting only once (73.7% females) compared to patients presenting multiple times (55.3% females; *Χ*^2^(1, *n* = 952) = 34.59; *p* < 0.001). 98.8% of former ICU patients and 96.6% of the patients with former inpatient treatment, versus 21.3% of the patients with former outpatient treatment, presented more than once. With regard to the presence of pre-existing conditions, significant differences could be seen in relation to chronic heart failure (*Χ*^2^ (1, *n* = 913) = 6.662; *p* = 0.010) and coronary artery disease (*Χ*^2^(1, *n* = 912) = 13.269; *p* < 0.001). This could not be demonstrated for the other pre-existing conditions analyzed (chronic lung diseases (*Χ*^2^(1, *n* = 919) = 3.157; *p* = 0.076), coagulation disorders (*Χ*^2^(1, *n* = 913) = 0.187; *p* = 0.665), chronic pain (*Χ*^2^(1, *n* = 912) = 2.76 *p* = 0.097) or mental disorders (*Χ*^2^(1, *n* = 914) = 0.006; *p* = 0.938)).

At the time of initial presentation, the post-COVID score was 21.4 points (SD = 10.2) for those presenting only once, compared to 20.2 points (SD = 10.8) for those presenting multiple times (*t*(950) = −1.84; *p* = 0.067). For symptom complexes presented at the time of initial presentation, significant differences between one-time and repeated presenters were seen only for the symptom complexes of fatigue (83.4% of one-time presenters; 75.9% of re-presenters; *Χ*^2^(1, *n* = 952) = 8.21; *p* = 0.004) and neurological impairments ailments (80.3% of one-time presenters; 69.6% of re-presenters; *Χ*^2^(1, *n* = 952) = 14.43; *p* < 0.001).

## 4. Discussion

This study presents the first real-world evidence on the readily applicable PCS score recently developed by Bahmer et al. [14]. The results show that disease progression is variable and that symptoms can still be present months after acute infection, as also noted by several previous studies [10,20].

There are significant differences between the cohort studied here and that of Bahmer et al. that must be taken into account when interpreting the results. While the cohort of Bahmer et al. was automatically contacted by public health authorities, irrespective of initial disease severity or symptom persistence, and study visits were scheduled at least 6 months after the confirmed infection with SARS-CoV-2, the patients of this study sought specific medical help in a specialized post-COVID outpatient clinic because of subjectively suffering from various complaints and functional limitations. Thus, there is a general motivational difference regarding visits to the study center, which may influence the results. Therefore, it was relevant to further verify the post-COVID score also in a clinically derived sample to identify potential gaps in the score and to illuminate its applicability in an outpatient setting with patients presenting based on their individual suffering. The possibility of using the post-COVID score also in international comparison is an option, as described above, because of the sample used. In addition, the patients of Bahmer et al. are predominantly non-vaccinated patients of the 1st and 2nd wave, which is why the availability of vaccine and medication does not play a role. One problem concerning generalizability of the score might be general demographics and pre-existing conditions (in terms of chronic inflammatory diseases), as this is different in the German study population compared with low- and middle-income countries. The validity and reliability of the post-COVID score used were covered by two elements: (1) replication was performed in two cohorts that produced comparable results; and (2) benchmarking of the post-COVID score was performed against an established quality of life instrument (EQ-5D-5L) [14]. In addition to Bahmer et al., there are other instruments that can classify the severity of post-COVID or its functional limitations, such as the modified COVID-19 Yorkshire Rehabilitation Scale (C19-YRSm) [21], COVID-19 Rehabilitation Needs Questionnaire (RehabNeQ) [18], or Post-COVID-19 Functional Status scale [22]. The evaluation is a retrospective analysis, so it was not possible to use these specific questionnaires, which must be completed by the patients themselves at the time of the visit. In addition, the simple use of Bahmer’s post-COVID score on symptom complexes provides a quick and easy overview of disease severity.

The results of the current study show that the disease course is highly variable, and that PCS symptom complexes differ significantly between patients treated in an post-COVID outpatient clinic. The majority of patients included in this registry had moderate or severe PCS. When looking at the individual presentation time points, the symptom complexes recorded in the PCS score were completely absent in 4–5% of the patients. It is interesting to note that in the subgroup of patients who repeatedly presented to the post-covalent outpatient clinic, the number of patients without symptom complexes reported in the PCS score increased to 7 by the third appointment (there was only one person at the first and second appointments). Re-presentation indicates that they continued to suffer from a relevant, at least subjective, burden of disease, e.g., limited activity and participation, and need for medical treatment. Therefore, greater inclusion of functional aspects that may contribute to impaired activity and participation should be considered for classification of post-COVID patients. [23,24,25]. Especially with regard to health-related quality of life, it has been shown that there are patients who have no limitations in this respect, although symptoms are present [3]. However, the vagueness in the currently valid definition does not always allow a simple and unambiguous assignment. Particularly in the case of an exacerbation of preexisting symptoms in the context of a known disease, the cause of the exacerbation is not always comprehensible [26].

In a potential expansion and specification of the definition of PCS, objectifying the previously mentioned symptoms is an important issue. Our analysis showed that symptoms such as fatigue and depression, as reported in standardized questionnaires at the time of initial presentation, correlated with a higher post-COVID score, whereas time since acute infection at the same time point did not seem to influence severity. Further investigation is needed into persistent fatigue, which occurred in more than 80% of patients in the cohort at all three time points, and is estimated to have an overall prevalence of approximately 23% after COVID-19 [20]. More importance should be given to possible causes, especially with regard to postinfectious immunological changes, but also to a particular susceptibility in the case of already limited resilience prior to the disease. A recently published guideline for further research on this phenomenon recommends that, in addition to standardized recording of fatigue, postexertional symptom exacerbation (PESE), also called postexertional malaise (PEM), should also be recorded [27]. If affected patients suffer from such episodes, therapeutic and rehabilitative measures must be individually adapted to the patient’s performance and should not be considered from the point of view of a possible deconditioning [28]. The use of strict pacing protocols in therapy showed an advantage regarding the incidence of PESE [29].

This underlines once again that the treatment of PCS must be individualized and, in addition to the classic established diagnostic and therapeutic pathways, multimodal interdisciplinary approaches are particularly necessary [30,31]. For this purpose, additionally to regional post-COVID centers, the already-existing structures, such as general practitioners, should be included in networks in order to treat all the symptoms and their development, and to develop therapy algorithms in a holistic approach [32,33,34]. Classification tools such as the PCS score investigated here might help to harmonize communication between network partners.

In contrast to other studies, our analysis did not show pronounced PCS symptoms in patients with intensive care treatment during acute COVID-19. Further, when considering preexisting conditions, only the presence of coagulation disorders or coronary artery disease showed a significant effect on the score [14,20]. Of note, outpatients had higher scores on the post-COVID score than ICU patients, although almost all inpatients presented to the outpatient clinic more than once. However, our analyses also showed that patients with a more severe acute course presented earlier to the post-COVID outpatient clinic. The reasons for this may be multiple. On the one hand, patients treated in an intensive care unit often receive acute rehabilitation, whereas outpatients receive rehabilitation only if symptoms persist and, in many cases, after actively asking their primary care physician [35]. An improvement in symptoms at the time of presentation could explain this. On the other hand, critically ill patients might feel more comfortable in the health care system and may search for contacts more quickly, especially in the case of post-intensive care syndrome [36]. Their satisfaction at having survived the serious illness may also mean that they complain less about residual symptoms that may be considered mild compared to the original illness. In addition, data from a cross-regional post-COVID outpatient clinic cannot conclusively answer this question, as affected patients with severe sequelae may not be able to go to such an outpatient clinic and, in turn, mildly affected patients may seek out care structures closer to their home, e.g., a general practitioner.

On this background, further analyses should follow, using the post-COVID score, to show the course of the disease in different subgroups as well as identifying possible predictors that could be relevant.

This study has some limitations. The study cohort here included patients presenting in a post-COVID outpatient clinic for treatment. Therefore, no conclusions can be drawn about the course and overall prevalence of all affected individuals. In addition, there is a lack of information on why patients did not present again or cancelled the appointment. If the reason was solely recovery, this would be a positive outlook, but it cannot be interpreted here. Moreover, patients with pre-existing comorbidities were not excluded. However, these could have an impact on the course and persistence of a PCS.

The intervals between re-presentation appointments varied, so that the temporal aspect can only be used to a limited extent for a holistic interpretation. Also, only limited reference could be made to the acute illness and the course. There is a lack of information about possible drug treatments or existing symptoms during the acute phase, which could have an influence on the further course of the disease.

Third, patients may have received different therapies between visits, such as physiotherapy or occupational therapy, which could have had a positive impact on reported symptoms. Because these therapies were provided in outpatient facilities close to the patients’ homes, and not in the post-COVID outpatient clinic, we do not have precise information on the intensity and frequency of the possible therapies received.

For further assessment of the course of PCS, the inclusion of possible therapeutic actions is necessary, and should be addressed in further investigations. A positive influence of individual rehabilitation, also within the framework of telemedical concepts, in order to also provide medical service for rural regions or to save patients with more severe limitations in travelling distances, has already been proven in various studies [37,38].

## 5. Conclusions

The severity of PCS as indicated by the post-COVID score in a cohort of patients presenting to a specialized post-COVID outpatient clinic is highly variable. Sex and some preexisting conditions appeared to influence symptomatology and severity, whereas treatment intensity during the acute phase of infection and the interval between acute COVID-19 and presentation to the clinic seemed to have little or no direct influence. Further detailed analyses of the longer-term course of the disease, including the possible effects of therapeutic interventions, should be performed. In this respect, the post-COVID score has proven to be applicable in a real-world setting and might qualify as potential endpoint in future clinical trials. The building of networks and the involvement of all health service providers is necessary in order to adequately treat affected patients adequately.

## Figures and Tables

**Table 1:** Pre-existing conditions, acute medical treatment, and duration since infection at the time points of presentation (*n* = number of patients).

		*N*	%
**Pre-Existing Conditions**			
Chronic lung disease (*n* = 919)	existing	132	14.4%
Mental disorders (*n* = 914)	existing	125	13.7%
Chronic pain (*n* = 912)	existing	46	5.0%
Coronary artery disease (*n* = 912)	existing	49	5.1%
Chronic heart failure (*n* = 913)	existing	40	4.2%
Coagulation disorder (*n* = 913)	existing	27	3.0%
**Acute medical treatment**			
Inpatient treatment (*n* = 952)	no	711	75.8%
	yes	148	15.5%
	yes, with ICU	82	8.6%
**Duration since infection at the time points of presentation**			
Duration since infection (1st *presentation*)	3–6 Mon	249	26.2%
	6–9 Mon	250	26.3%
	9–12 Mon	178	18.7%
	12–15 Mon	149	15.7%
	15–18 Mon	81	8.5%
	18–24 Mon	42	4.4%
	>24 Mon	3	0.3%
Duration since infection (1st *re-presentation*)	3–6 Mon	1	0.3%
	6–9 Mon	67	17.7%
	9–12 Mon	97	25.6%
	12–15 Mon	99	26.1%
	15–18 Mon	66	17.4%
	18–24 Mon	46	12.1%
	>24 Mon	3	0.8%
Duration since infection (2nd *re-presentation*)	3–6 Mon	0	0.0%
	6–9 Mon	0	0.0%
	9–12 Mon	8	6.2%
	12–15 Mon	39	30.2%
	15–18 Mon	40	31.0%
	18–24 Mon	33	25.6%
	>24 Mon	9	7.0%

**Table 2:** Existing symptom complexes at the time points of presentation (*n* = number of patients).

		1st Presentation (*n* = 952)		1st Re-Presentation (*n* = 379)		2nd Re-Presentation (*n* = 129)	
		*n*	%	*n*	%	*n*	%
**Symptom complexes**							
Fatigue	existing	766	80.5%	304	80.2%	106	82.2%
Neurological ailments	existing	724	76.1%	290	76.5%	104	80.6%
Exercise intolerance	existing	512	53.8%	176	46.4%	69	53.5%
Joint and muscle pain	existing	400	42.0%	206	54.4%	72	55.8%
Sleeping disturbance	existing	364	38.2%	178	47.0%	63	48.8%
Chemosensory deficits	existing	281	29.5%	104	27.4%	26	20.2%
Gastrointestinal ailments	existing	141	14.8%	75	19.8%	35	27.1%
Cough, wheezing	existing	127	13.3%	63	16.6%	24	18.6%
Chest pain	existing	75	7.9%	22	5.8%	6	4.7%
ENT ailments	existing	67	7.0%	40	10.6%	15	11.6%
Dermatological ailments	existing	66	6.9%	25	6.6%	5	3,9%
Infection signs	existing	14	1.5%	3	0.8%	2	1.6%

## Data Availability

The data presented in this study are available upon reasonable request from the corresponding author.
